# Children’s representations of the COVID-19 lockdown and pandemic through drawings

**DOI:** 10.3389/fpsyg.2022.960893

**Published:** 2022-08-24

**Authors:** Alessia Cornaggia, Federica Bianco, Gabriella Gilli, Antonella Marchetti, Davide Massaro, Ilaria Castelli

**Affiliations:** ^1^Department of Psychology, Catholic University of Sacred Heart, Milan, Italy; ^2^Department of Human and Social Sciences, University of Bergamo, Bergamo, Italy

**Keywords:** children drawings, child development, COVID-19, emotions, relationship, mindreading abilities

## Abstract

The COVID-19 pandemic and the measures to face it have placed children and their caregivers in front of many challenges that could represent sources of stress. This work aims to explore the point of view of children through drawing, as a spontaneous means of expression, relating it to parents’ perceptions of children’s difficulties, strengths, and mentalization skills. The sample consists of 18 children (mean age = 8.22, SD = 1.79). Parents were asked to complete: a socio-demographic questionnaire with information on the impact of COVID-19 on the family, the Strengths and Difficulties Questionnaire, and the Everyday Mindreading Scale. Children were asked to draw three moments: “Before” the pandemic, “During” the lockdown, and “After,” when the COVID-19 will be passed. The drawings were coded by constructing a content and expressive analysis grid, adapting coding systems found in the literature. Data were collected at the beginning of the summer of 2020, just after the first lockdown period (from March to May 2020 in Italy). The results of the present work are in line with previous studies that reported experiences of wellbeing and tranquility of children in time spent at home with family during the pandemic. From the drawings emerges that children feel sufficiently able to master the situation, as reflected by including themselves in drawings and providing many details of the house in “During” drawings. The literature also reports a feeling of sadness/loneliness caused by the lack of friends, an element that we also find in the tendency to represent friends significantly more in the drawings concerning the future. Some contents of drawings (inclusion of friends, relatives, and parents) appeared associated with emotional, interpersonal, and mentalizing abilities of children, as perceived by parents. Exploring children’s representations of a stressful event like the pandemic through drawings allows to focus both on their difficulties and on their resources, with useful implications for the educational support.

## Introduction

The COVID-19 disease, declared as a pandemic in March 2020 by the World Health Organization (World Health Organization [WHO], 2020), has spread throughout the world radically changing human habits, relationships, and contexts of life. In addition to the crisis in the socio-sanitary systems and to the impact on physical health, COVID-19 brought about a whole series of psychosocial implications that affected the majority of aspects of people’s lives (Adıbelli and Sümen, 2020; Di Giorgio et al., 2020; Marchetti et al., 2020; Petrocchi et al., 2020; Ghanamah and Eghbaria-Ghanamah, 2021; Mantovani et al., 2021). Italy was the first European country that had to face the emergency, so the Government introduced from March to May 2020 stringent measures to contain the epidemic, i.e., a general lockdown. Children and adolescents represented about 16% of the Italian population that during the pandemic could no longer go to school and no longer meet their classmates and teachers (Caffo et al., 2020): in short, they saw their daily life suddenly overturned. Therefore, despite most infected children being asymptomatic or presenting mild clinical manifestations (Jiao et al., 2020), some studies investigated, at different levels and in different ways, the impact of COVID-19 on children’s lives. In particular, risk factors included family disruption due to illness or death, financial instability tied to job loss, and educational disruptions as a result of the closures of early child care facilities and schools, as well as transitions to online learning (United Nations Human Rights Office, 2020). Moreover, the fear of infection, frustration and boredom, circulation of misinformation, and limited access to reliable sources of information increased the stress level (Cluver et al., 2020; Presti et al., 2020; Ren et al., 2020).

Given the hard and various challenges that children had to face during this prolonged emergency (Pascal and Bertram, 2021; Samji et al., 2022), it could be relevant to consider both immediate and long-term effects on child development across developmental domains (Benner and Mistry, 2020).

The social domain was the most evidently impacted: with the closure of schools and sport-leisure facilities, children lost not only the opportunity to learn from their peers, teachers, and educators (Haleemunnissa et al., 2021) but also the sharing of experiences, learnings, and feelings that were difficult to achieve through a screen. Moreover, the economic and pandemic-specific factors increased parents’ stress and undermined the quality of relationships among family members, including marital, parent-child, and sibling relationships (Prime et al., 2020). Another relevant loss for children was the support of important caregivers like grandparents, who often, especially in Italy, play a crucial role in family welfare—taking care of grandchildren while parents are at work—thus becoming fundamental caregivers for children (Caffo et al., 2020).

Children’s behavioral development (Dray et al., 2017; Clark et al., 2020; Wang et al., 2020) was challenged by home confinement: indeed, sudden changes in living habits precluded children from the possibility to relate outside the family, such as practicing physical activities and especially experiencing independence and autonomy in educational contexts different from the family one, such as schools, sports teams, recreational centers, and so on (Brazendale et al., 2017). All these elements became risk factors for the emergence of hyperactivity, conduct problems, sleeping and eating disorders, and psychological distress in children (Petrocchi et al., 2020; Wang et al., 2020; Bianco et al., 2021; Liu et al., 2021). In addition, health emergency widened risk factors already existing in families and fragile social contexts before the advent of COVID-19 (Carneiro et al., 2016; Liu et al., 2021).

Regarding the emotional domain, some authors (Brooks et al., 2020; Jiao et al., 2020; Jiloha, 2020) pointed out the negative emotional effects created by the confinement situation. The adverse impact on the emotional sphere became more severe as the duration of the confinement increased (Brooks et al., 2020). Emotions, such as fear and sadness, were not only linked to the lockdown, but also to the virus itself, as children were concerned about the uncertainty and unfamiliarity of the situation (Idoiaga Mondragon et al., 2021). The study conducted by Orgilés et al. (2020) pointed out a worsening in children’s emotional state and behavior, especially difficulties in concentration, boredom, irritability, and loneliness (Orgilés et al., 2020). In addition to the specific psychological effects of the lockdown, great concern was created by the uncertainty about the personal and global effects of COVID-19 (Brooks et al., 2020).

In such a complex emergency scenario, potentially contributing to traumatic situations, children should have the possibility to attribute meaning to the changes that they observe around them or that they personally undergo (Stein et al., 2009). Psychological literature has long stated that “*Children are and must be seen as active in the construction and determination of their own social lives, the lives of those around them and of the societies in which they live*” and not merely as “*passive subjects of social structures and processes*” (Prout and James, 1990, p. 8). It is, therefore, crucial to directly consider children’s point of view on the radical changes caused by the pandemic through instruments that are as close as possible to their natural and spontaneous expressive and communicative mode. Drawings seem the most appropriate method to fulfill this goal. First of all, the use of drawings in psychological research has a long tradition both in developmental and clinical psychology (e.g., Machover, 1949; Royer, 1989; Malchiodi, 1998, 2001, 2008; Eaton, 2007; Romano, 2010), and it is supported by the age-appropriateness of the method “*drawing is a natural mode of expression for children age 5–11*” (Koppitz, 1968, as cited in Fury et al., 1997, p. 1154). Secondly, the request to draw a life experience enables researchers to collect information that is unlikely to emerge through verbal and observational methods (Pinto, 2016). In fact, drawing may represent, especially for younger children, a form of communication that is easier to access and has fewer constraints than verbal communication. Moreover, this communication medium can bring out implicit knowledge and content regarding experienced situations, as well as emotional aspects that might be too difficult to express through the verbal channel (Pinto, 2016). In addition, the realization of a drawing is a task that the child can carry out independently and, compared to other methods, that allows limiting the influence of the adult and the researcher (Thomas and Jolley, 1998). Finally, besides exercising and expressing imagination and creativity, the benefits that children could derive from the activity of drawing are known to be various and at different levels (Barnes, 2002; Burkitt et al., 2010; Hetland et al., 2013): cognitive (visual thinking, observation, analysis, problem-solving), behavioral (perseverance, experimentation, and reflection) and also emotional, because creative activities as drawing are known to allow children to express and share their emotions, including anger and fear that situations like a pandemic could elicit (Adıbelli and Sümen, 2020). In particular, in considering a drawing we could distinguish two levels: the representational one and the expressive one (Brechet and Jolley, 2014). Children’s experiences could influence the use of specific and different sources of inspiration (Rose and Jolley, 2020) and children’s perspectives could be communicated in a drawing through different channels: literal, content, and abstract expression (Brechet and Jolley, 2014).

For all the above reasons, the adoption of drawings has been a common methodological procedure in previous studies investigating the traumatic impact on children of events such as earthquakes or wars, with the double valence of a diagnostic and a therapeutic tool (Malchiodi, 2001, 2008; Romano, 2009, 2010). Regarding the graphical tool, it has already been repeatedly used in taking care of children victims of natural disasters (Malchiodi, 2001, 2008; Crocq et al., 2005; Eaton, 2007; Orr, 2007), as it allows to express emotions and events that were too painful to be told, in a structured way. The expression of feelings and thoughts through the graphic object is less explicit and therefore less threatening than the real word (Steele et al., 1995; Malchiodi, 1997). Through drawings, children are able to reprocess traumatic emotions and thoughts, contextualize the event lived in the history of their lives, and give it a new meaning, thus facilitating the processes of elaboration (Crocq et al., 2007; Hariki, 2007). The act of drawing enables children to discover and organize their impressions by following the inspirations of their own emotionality (Quaglia, 2003). Furthermore, what children draw with care could represent their positive affective tendency, and also omissions in the drawings should be considered informative because they could be an indication of an intolerable reality for the child (Quaglia and Saglione, 1990). Through drawing it is therefore possible to achieve internal cohesion, combining perceptive, affective, driving, and narrative aspects of lived life experiences, in order to create a “unified mental entity” (Royer, 1995, p. 15). Studies regarding prisoners of war camps (Volavkova, 1978) showed that in emergencies the drawing was first of all a spontaneous instrument of survival. Therefore, it is important that children in an emergency or traumatic situation are left free to draw what they want, in order to give meaning to the lived experience (Kalmanowitz and Lloyd, 1999; Al-Krenawi and Slater, 2007). In other studies, instead, children were given a more specific task, for example, the request to draw what happened or to represent themselves during the traumatic event, and/or the body of the victim (Malchiodi, 1998). In traumatic contexts, a widely used instrument is the “Test de trois dessins: avant, pendant et avenir” (Crocq et al., 2005). The technique of splitting time into three moments was introduced by Brauner and Brauner (1976) and it was subsequently resumed in other studies regarding the traumatic impact of wars and earthquakes on children, even if with some differences in the task (Bonnet, 1994; Nebout and Nebout, 2000; Crocq et al., 2002, 2005; Coq and Cremniter, 2004). The structure of the instrument with three points of time allows going deep inside into one’s own memory, in order to seek continuity of life between the past and the future, encouraging one to set contact with the world and with others, thus promoting the reintegration of the subject in context. For these reasons, in this work, children’s experiences were collected by referring to three moments: “Before” the pandemic advent, “During” the lockdown experience, and in the “Future,” when the COVID-19 emergency will be over.

The first general objective of this study was to explore the representations of children related to COVID-19 through drawings and verbal comments on their own drawings. We aimed to observe which themes and emotional connotations emerged spontaneously in drawings, in order to understand which meanings, both in terms of difficulties and of resources, children constructed about the COVID-19 experience. Therefore, in this work drawings were considered first of all as a communicative act (Pinto, 2016), an instrument that children could use to express tacit or explicit contents and to share meanings with another person. We hypothesized that, given the emergency period, negative feelings would emerge, both at expressive and content levels, for example through themes related to the precautionary measures contrasting the infection and through the inclusion of negative connotated expressions. However, we did not *a priori* exclude the presence of positively connoted experiences as, for example, spending more time with the family. Moreover, at the expressive level, we expected that older children would have realized drawings with more expressive elements (Jolley et al., 2004).

Secondly, thanks to the request to produce drawings concerning three different moments (before, during, and in the future, after the pandemic experience), the present study had the purpose to compare representations emerging from three different time points, both at a content level and at an expressive level. To this second aim, we adapted the “Test de trois dessins: avant, pendant et avenir” (Crocq et al., 2005) to make it suitable for the specific features of the pandemic emergency and the specificity of the procedure and the context of data collection. We expected that the memories of the past and the image of the future would have included the representation of multiple people, external spaces, experienced with positive connotations, while the lockdown period would be the one most characterized by the home context and elements that refer to negative experiences. However, negative connotations could also emerge in drawings concerning the past, reflecting melancholic memory of people or experiences that are no longer here, or in the future in the form of fear and dread outweighing hope.

Besides children, another level of analysis included the parents’ point of view on their children (third aim of the study). Parents compiled an online set of questionnaires that investigated the impact of COVID-19 in family life, and a report on some social, behavioral, and emotional aspects of their own children’s experience and development. The aim was to investigate possible connections between the content and expressivity of drawings on the one hand, and, on the other hand, the difficulties encountered by children during the pandemic, their resources, and their mentalizing abilities to cope with it, as reported by parents in questionnaires. It was expected that there may have been connections between the expressive connotations of the drawings, the level of mentalistic ability and emotional difficulties detected by parents in their children, as previous literature suggests that the emotional comprehension and the expressivity of drawings are connected (Brechet and Jolley, 2014). In addition, difficulties at the relational level could be related to the number and the typology of people included in the drawings in agreement with the idea that children tend to include in their drawings what and who is perceived as reassuring and trusted (Quaglia and Saglione, 1990).

## Materials and methods

### Procedure

The Ethical Committee for Research of the University of Bergamo gave its approval for the research (Report No. 7 of 22nd May 2020) and all requirements of the ethical guidelines were respected (World Medical Association, 2008; AIP, 2015; American Psychological Association [APA], 2017). The recruitment was done through main social media platforms (i.e., WhatsApp and Facebook) where a brief presentation of the research was inserted, inviting interested parents to reply to the communication in order to be contacted by one of the researchers and to receive all the instructions for participation. In the ads, we reported the required age of the participant, the general aim of exploring children’s experience of COVID-19, and the commitment required (online questionnaire, children’s drawings, and audio comments). Parents interested in the study were 22 and were all contacted telephonically by one of the researchers to describe the study, explain the fundamental steps necessary to participate, and propose participation for themselves and their child/children. On this occasion, parents could make questions to the researcher, and they received the researcher’s contacts for further pieces of information if needed. All parents interested to participate were provided with a link to the online platform of the study. The expression of informed consent was a prerequisite to participate in the study: the document was available for parents’ completion in the online link. All participants could withdraw at any time. After telephone contact, we had confirmation of acceptance to take part in the study for 18 children. The online form for parents included three tasks: a socio-demographic questionnaire, the Strength and Difficulties Questionnaire (Goodman, 1997, 2001), and the Everyday Mindreading Scale (Peterson et al., 2009). Then, parents were asked to propose their children to realize three drawings related to the COVID-19 emergence and subsequently to record a verbal parent-child interaction about the three drawings. The request for recording comments was due to ensure that the researcher could understand all the elements of the drawing. Indeed, at a methodological level, the audio contents allow collecting more information about the representations, intending to comprehend also the meanings attributed to the drawings directly by authors, thus avoiding the risk of incurring over-interpretations. Furthermore, since the parents and not the researcher interacted with the children, the audio request could also represent a precious opportunity for the parent-child dyadic system to dialog, discuss, share emotions and meanings elicited from this period of emergency in which, as already mentioned, the whole family system was faced with new challenges and redefined itself according to the new situation. Due to the ongoing health emergency in the summer of 2020 when data were collected, the administration was necessarily done remotely and the direct interlocutors of the children were the parents. We provided parents with some brief instructions to help them in the presentation of the activity to their children and we recommended them not to force in any way the realization of drawings and comments of their children. A time of about 1 week was given to children in order to choose the most suitable time for their drawings and to proceed as spontaneously as possible without fatigue. We required to complete all three drawings in the same session. Inclusion criteria included having children in middle childhood, who speak Italian fluently and live in Italy. As exclusion criteria, we considered having developmental disorders.

### Participants

The research involved 18 children (8 males and 10 females), with an age range from 5 to 12 years (*M* = 8.22, *SD* = 1.79). Parents involved (4 males and 14 females) had an age range from 35 to 49 years (*M* = 41, *SD* = 3.91), with different educational qualification: 50% secondary school qualification, 11.1% Bachelor’s degree, 22.2% Master’s degree, 16.7% postgraduate specialization.

### Measures

The present study combined qualitative and cross-sectional quantitative measures, indeed in drawings we considered both qualitative and quantitative indicators, and questionnaires gave us quantitative data. Children were asked to produce three drawings on three moments of the COVID-19 pandemic and to orally explain the content of their drawings. The request for both the drawings and the audio report was made by parents to their own children.

Parents completed three questionnaires: a Socio-Demographic form, the Strength and Difficulties Questionnaire (Goodman, 1997, 2001), and the Everyday Mindreading Scale (Peterson et al., 2009).

### Children’s drawings and oral explanations on drawings

The graphical representations were investigated through an adaption of the “Test de trois dessins: avant, pendant et avenir” (Crocq et al., 2005), an instrument used previously in literature to explore the impact of traumatic events such as wars and earthquakes on children’s representations (Crocq et al., 2005; Giordano et al., 2015). In the classical version, the task was composed of three parts requiring children to draw themselves, their family, and their house before, during and after the potentially traumatic event, i.e., war or earthquake. In the present study, some adaptations were applied due to the different types of emergences involved (i.e., COVID-19 pandemic), and to the different settings of administration (at home with parents). Parents were asked to make available to the child white sheets, pencils, colored pencils, and to propose them to realize three drawings related to different moments in the timeline of the COVID-19 spread and lockdown period: *a day that they remember before the coronavirus arrived (“BEFORE drawing”), a day among those they have lived during the lockdown (“DURING drawing”), a day of the future, when the coronavirus will be defeated (“FUTURE drawing”).* Subsequently, parents asked their children to explain their drawings, and motived the necessity to audio-record their narratives to ensure that the researcher could understand all the elements of the drawing. Parents were also advised not to force the child to draw or record the comments on the drawings in any way, to avoid intervening in the realization of the drawings and to remember to their children that there was no evaluation, but only an interest to know what children were thinking about the COVID-19 emergence.

For the coding procedure, we followed previous literature (Picard et al., 2007; Giordano et al., 2015), but at the same time, we constructed a specific grid (see Table 1) to identify two levels of information: the content of the representations and the expressive connotations of drawings. On one side, we used some indices from previous work that investigated the traumatic impact of natural disasters (Giordano et al., 2015), such as the presence of the house, the representation of themselves, parents, or others. In addition, we evaluated if children included details of internal vs. external spaces, the representation of pandemic characteristic elements (e.g., masks, graphical representation of the virus, the slogan “*Everything will be ok*”…),^1^ of their school or of online lessons. At the content level, we distinguished indices related to the richness of pictures and others that detected the type of content. A general index of richness was represented by the number of elements in the drawing (object, people, nature…). For the representations of people, indices of richness were the number of human figures included, and the category of people represented (parents, relatives, friends, strangers, …). In evaluating the expressive strategies used by children to convey the positive or negative connotation of drawings, we moved on from the theorical framework of Jolley (2010) and we adapted some indices used in the previous work of Picard et al. (2007), aka objects and natural elements that suggest happiness or sadness. We also maintained some indices that were used both in Picard et al. (2007) and in Giordano et al. (2015), i.e., the number of colors, and the presence of happy or sad facial expressions. Moreover, we included at the expressive level the presence of indicators of movement. Specifically, our index of movement representation constituted an integration of the index concerning body position (Picard et al., 2007) and narrative elements (Giordano et al., 2015) related to dynamism. In detail, we considered as presence of movement in the drawings those elements of dynamisms were either deducted from the body position and gestures of characters or from the explanation children gave to their drawings. Drawings were coded independently by two of the authors, that subsequently discussed mismatches in coding, in order to provide a joint decision on the codification to be assigned.

**TABLE 1:** Indices used in coding drawings.

	Indices	Coding
Content		
Typology	Inclusion of COVID-19 references	1 = Absent 2 = Present
	Inclusion of school references	1 = Absent 2 = Present
	Themselves	1 = Absent 2 = Present
	Parents	1 = Absent 2 = Present
	Relatives	1 = Absent 2 = Present
	Friends	1 = Absent 2 = Present
	Other people	1 = Absent 2 = Present
	House	1 = Absent 2 = Present
	Space of the house	1 = Internal 2 = External
Richness	Colors	Number
	Elements	Number
	People	Number
	Details of the house	1 = Absent 2 = Present
Expressive connotation		
	Positive natural elements (sun, rainbow, flowers…)	1 = Absent 2 = Present
	Negative natural elements (clouds or rain, spiders, snakes, sickly leaves or flowers …)	1 = Absent 2 = Present
	Positive objects (gifts, details on clothes, hearts…)	1 = Absent 2 = Present
	Negative objects (broken objects, empty cavities…)	1 = Absent 2 = Present
	Facial expression of happiness	1 = Absent 2 = Present
	Facial expression of sadness	1 = Absent 2 = Present
	Representation of movement	1 = Absent 2 = Present

### Socio-demographic form

Parents were asked to complete a questionnaire based on the socio-demographic form used for the previous works by Petrocchi et al. (2020) and Bianco et al. (2021), with some differences due to the specificity of the aims of each work. It was composed of 14 questions about: socio-demographical data (age and gender of parent and child, parent’s education level, family residence, presence/absence of development disorders and fluency in the Italian language of the child, changes in socio-economic status due to pandemic), the exposure to COVID-19 (if they relatives and/or their friends were positive for the virus infection or manifested correlated symptoms and whether someone died because of COVID-19), the presence of garden or terraces in their home and the people with whom the child had spent the quarantine.

### Strength and difficulties questionnaire

The parents’ perception of their children’s difficulties and/or resources was investigated through the Strength and Difficulties Questionnaire (SDQ; Goodman, 1997, 2001). The original instrument was composed of 25 items grouped in five subscales: *Hyperactivity, Emotional Symptoms, Conduct Problems, Peer Problems*, and *Prosocial Scale*. In this work two items of the *Peer Problems Scale* were removed (“Picked on or bullied by other children” and “Generally liked by other children”), because in the specific period of the lockdown these aspects were not evaluable.^2^ Each item could be marked “not true,” “somewhat true” or “certainly true.” The items that express difficulties were scored 1 for “not true,” 2 for “somewhat true,” and 3 for “certainly true.” The items that express a strength point were scored 3 for “not true,” 2 for “somewhat true,” and 1 for “certainly true.” The scores for *Hyperactivity, Emotional Symptoms, Conduct Problems*, and *Peer Problems* were summed to generate a total difficulties score ranging from 18 to 54. A higher total score indicated a major level of difficulty and this was true also for subscales, except for the *Prosocial Scale* which was not incorporated in the reverse direction into the total difficulties score, as indicated in the guidelines of the questionnaire. In this subscale, the range was from 5 to 15, with a higher score indicating a major presence of prosocial behavior.

### Everyday mindreading scale

The parents’ attribution of mentalizing abilities to their children was measured through the Everyday Mindreading Scale (Peterson et al., 2009). This instrument was composed of 8 items: six items concern children’s attitudes that reflect their difficulty in considering others’ perspectives, whereas two items relate to the children’s ability to adapt their behavior to the context and to take into account others’ emotional expressions. For each statement parents had to rate their own child’s difficulty using a 5-point scale where 1 was “not true” and 5 “completely true.” We reverse-scored positive items so that a higher score on each item of the scale reflected more difficulty (total scores ranging from 8 to 40).

### Data analysis

The collected data have been analyzed using the SPSS statistical software Version 25. The sample size and the distribution of variables led us to use non-parametric analysis techniques. Single sample tests (binomial and chi-square) were executed to examine the distribution of frequencies of indices in the three groups of drawings: “Before drawing,” “During drawing,” and “Future drawing.” The distribution of each variable in the coding grid within the three tests was compared by testing non-parametric hypotheses on repeated measurements. The Friedman test was used for quantitative analyses, Cochran *Q*-test was used in the case of qualitative binary variables. In presence of significant differences between the distribution of the variables, three “pair” comparisons were carried out to investigate the nature of the significant differences that exist. Three “pair” comparisons were performed together in a single phase, thus a Bonferroni correction for multiple tests was considered and *p*-values were compared with a significance level α = 0.05. Mann-Whitney test was used to compare qualitative drawing indicators and the scores of the parents’ questionnaires.

## Results

Table 2 reports some specific characteristics of the sample that describe the experience of the lockdown of children and also the impact of the pandemic on their family life. In particular, it seems important to emphasize the fact that all participants had an outdoor space during the lockdown, most families did not experience deaths due to COVID-19 and more than half of the sample did not even encounter any positive cases either in the family or in the circle of friends. All children spent the lockdown in the family with at least one parent and most of children also with brothers or sisters. Finally, the involved families tended not to have experienced significant changes in socio-economic status due to the pandemic.

**TABLE 2:** Sample description of COVID-19 related information.

Collected information	Percentage
Features of the house	16.7% garden 16.7% terrace 66.7% both of them 0% none of them
Pandemic experience in family	66.7% no cases of COVID-19 positivity 5.6% someone had compatible symptoms but without doing a swab 27.8% cases of positivity in families 16.7% deaths caused by COVID-19
Pandemic experience in friends	60% no cases of COVID-19 positivity 40% cases of positivity in friends
Cohabiting during the lockdown	66.7% parents plus brothers/sisters 22.2% one or both parents 11.2% also grandparents
Changes in socio-economic status due to pandemic	94.4% absence 5.6% presence

With reference to the first and most exploratory objective of this paper, Tables 3, 4 show a descriptive overview of the main features of the drawings. Overall, there was a presence of contents and indicators with a positive connotation and a paucity of negative connotations in drawings. The one-sample binomial test showed that in the representations of all the three moments considered, children included themselves in drawings to a greater degree than expected (*p* = 0.001 “Before”; *p* = 0.008 “During” and “Future”). Comparisons based on age showed a significant effect on positive connotated objects, such as pictures of hearts, presents, clothes details, *U* (*N* Absence = 13, *N* Presence = 5) = 55.00, *z* = 2.22, *p* = 0.026. Older children (*Mdn* = 10.15) tended to represent these types of objects to a greater extent than younger children (*Mdn* = 7.44), that generally did not include them in their drawings of the future.

**TABLE 3:** Descriptive statistics on quantitative drawing indices.

Index	Before drawings				During drawings				Future drawings			
			
	*Min*	*Max*	*M*	*SD*	*Min*	*Max*	*M*	*SD*	*Min*	*Max*	*M*	*SD*
Number of colors	1	13	6.94	3.52	1	12	6.22	3.40	1	11	5.89	3.41
Number of people	0	9	2.78	2.49	0	6	1.89	1.68	0	15	3.44	3.94
Number of elements	1	10	4.44	2.48	1	12	5.50	2.77	1	8	3.83	2.01

**TABLE 4:** Frequencies of qualitative drawing indices.

Index	Before drawings Percentage	During drawings Percentage	Future drawings Percentage
Representation of the house	16.7	72.2	5.6
Details of the house	16.7	66.7	5.6
House as internal space	16.7	50	5.6
Representation of themselves	88.9	83.3	83.3
Representation of parents	16.7	27.8	16.7
Representation of relatives	16.7	38.9	16.7
Representation of friends	44.4	5.6	55.6
Happy expression	61.1	38.9	72.2
Sad expression	–	11.1	–
Positive natural elements	55.6	44.4	50
Negative natural elements	–	5.6	–
Objects with positive connotation	38.9	22.2	27.8
Objects with negative connotation	–	–	5.6
Imaginary contents	5.6	11.1	–
Elements that suggest movement	61.1	44.4	55.6
School referred elements	33.3	16.7	33.3
COVID referred elements	–	22.2	16.7

Concerning the second aim of this work, multiple use of colors and the inclusion of facial expressions of happiness were more frequently present in “Before” and “Future” drawings, but also in “During” drawings, where sadness was included by few children and solely in the period of strict home confinement (see Table 4). Similarly, there were positive connotated natural elements (i.e., sun, flowers, rainbow…) in “Before” and “Future” drawings, and even in the lockdown representation. The presence of movement, which once again was prevalent in “Before” and “Future” drawings, was also present in “During” drawings when the possibility of movement activities was limited, as illustrated in Table 4. Representations of the period before and after the lockdown were joined by some absences in pictures: these drawings generally did not include the representation of home (*p* = 0.008 “Before” and *p* < 0.001 “Future”), parents (*p* = 0.008 “Before” and “Future”) and other relatives (*p* = 0.008 “Before” and “Future”), compared with the expected distribution. If instead, we consider the drawings relating to the lockdown period, friends and references to school (also as online learning) were generally absent, but also there were not many elements that refer directly to COVID-19. Table 5 shows the comparisons between the distributions of the drawings variables at the three moments considered. As seen above, the house was generally not included in drawings representing the past and future. In line with this first evidence, a significant difference emerged also between representations of these two moments and the pictures concerning the period during the lockdown, where houses were more present, χ^2^(2) = 19.08, *p* = 0.000. Moreover, houses represented in drawings regarding the months of closures and home confinement were more detailed as compared with houses included in pictures of a day that children remember before the advent of the pandemic and their imagination of a day in the future when the emergency related to COVID-19 will be over, χ^2^(2) = 17.17, *p* = 0.000. Another significant difference, that once again concerns the period of lockdown in contrast with the other moments, was the representation of friends, χ^2^(2) = 10.31, *p* = 0.006, that were generally excluded from drawings representing the more critical phase of the emergency and instead were more depicted in the “Future” drawings.

**TABLE 5:** Significant differences in the distribution of variables within the three tests.

Index	Drawing			Comparisons		
		
	1	2	3	1–2	2–3	1–3
Presence of the house	3 (16.7%)	13 (72.2%)	1 (5.6%)	0.002*	0.000**	1.000
Inclusion of details of the house	3 (16.7%)	12 (66.7%)	1 (5.6%)	0.004*	0.000**	1.000
Presence of friends	8 (44.4%)	1 (5.6%)	10 (55.6%)	0.052	0.007*	1.000

1 = BEFORE drawing. 2 = DURING drawing. 3 = FUTURE drawing.

*p < 0.05. **p < 0.01.

As expected, in agreement with the third objective, there were some significant associations between indicators in drawings and scores in questionnaires. As far as regards the parents’ perception of mentalizing abilities of their children, it emerged that children that did not include their parents in the “Before” drawing had, according to their parents’ point of view, less difficulty in taking into account the mental states of others (*Mdn* = 12), in comparison with children that included their parents in drawing (*Mdn* = 17), *U* (*N* Absence = 15, *N* Presence = 3) = 41.00, *z* = 2.22, *p* = 0.027. In relation to the particular aspects identified by the subscales of SDQ, our results highlighted significant association with “Problems with peers”: children that tended to include relatives in drawings regarding the period before the pandemic had lesser relational difficulties with peers according to their parent’s evaluation (*Mdn* = 3), whereas children that obtained higher scores in this scale (*Mdn* = 4) tended to exclude relatives from their pictures *U* (*N* Absence = 15, *N* Presence = 3) = 4.50, *z* = −2.25, *p* = 0.027. It is necessary to point out that the index “Presence of relatives” also includes siblings. Finally, the scores in the subscale that measure difficulties in emotion regulation showed multiple significative associations with different drawings’ indicators. Concerning the representations of the period before the lockdown, children that represented friends in their drawings had lower levels of difficulty, referred by parents, in the emotional area (*Mdn* = 5), while children with higher emotional difficulties (*Mdn* = 8) tended to not include friends in their pictures, *U* (*N* Absence = 10, *N* Presence = 8) = 15.00, *z* = −2.29, *p* = 0.027. Moreover, children that included references to school in drawings had lower reported difficulties in this subscale (*Mdn* = 5), compared with children that did not draw any elements linked with school context (*Mdn* = 8) in representation of their past experience *U* (*N* Absence = 12, *N* Presence = 6) = 9.00, *z* = −2.60, *p* = 0.01. An interesting result emerged from the comparisons between the emotional subscale and the happy facial expression in drawings related to the period of quarantine (*H* (2) = 7.89, *p* = 0.023): children whose parents reported lower levels of emotional difficulties (*Mdn* = 5) tended to represent an expression of happiness, instead children that did not include happy faces received higher scores in the emotional difficulties scale from parents (*Mdn* = 8.50).

## Discussion

Exploring children’s representations of an emergency experience that upset their everyday life has highlighted a generally encouraging picture in considering the impact of COVID-19 on the children involved in this study. However, it is important to underline some characteristics of the sample that may have influenced children’s representations and, in general, the ability of families to deploy resources to limit the negative impact of the pandemic on their lives. The availability of outdoor spaces where children could spend time, a limited direct experience of COVID-19 and a limited impact on the socio-economic status of the families, could have affected the ways children involved in our study perceived and lived the pandemic.

Addressing the first aim of our study, in this section we will provide a discussion on the representations of children of COVID-19 pandemic, *via* the themes and expression connotations emerging from drawings. Conversely to what expected, we did not retrieve a high frequency of negative connotations in drawings. This result is in line with the one retrieved by Adıbelli and Sümen (2020) regarding children’s positive self-reported quality of life, despite home confinement. However, we should also remember, in our interpretation of this result, that our sample was mostly made by “privileged” families compared to the average of Italian families, even if they lived in a region severely affected by COVID-19 like Lombardy (Mantovani et al., 2021). In a similar direction, we found that children tended to insert themselves in drawings, suggesting that they feel able to cope with the situation also integrating the lockdown experience in the narration/representation of their life story. As pointed out by Masten (2021), a child’s reaction to an emergency largely depends on the capabilities that the family can allocate to cope with it, which in turn are influenced by other interconnected systems, first and foremost community support. The results of a work by Mantovani et al. (2021) supported this aspect, showing the importance of the so-called “systemic resilience” (Bronfenbrenner, 1979) characterized by the acceptance of limitations by children and by the effectiveness of parents in addressing the challenges posed by the pandemic. In line with literature, at the expressive level, we retrieved an effect of age on the inclusion of positive connotated objects in drawings (e.g., gifts, details on clothes, hearts…). The literature on children’s drawings suggests, indeed, that generally expressive ratings increase with age, but they are often significantly lower for sad drawings (Jolley et al., 2004). Furthermore, expressive contents are linked with emotional comprehension abilities and with knowledge about the situational determinants of emotions (Brechet and Jolley, 2014). Therefore, this age difference could be brought back to the development of expressive strategies used in drawings which has emerged despite the limited number of participants.

In response to the second aim of the study there are some results to highlight on the comparison of the representations of the three moments. All children have completed the drawings of all three moments, and the presence of a limited number of significant differences between “Before, During and Future drawings” could suggest linearity and harmony in the perception of their experience, despite the sudden changes imposed by COVID-19 and the consequent social restrictions. However, the observed frequencies of drawings indicators showed in particular an interesting result, that is the absence of parents especially in “Before” and “Future” drawings. This result goes in line with previous studies on the traumatic impact of natural disasters (Giordano et al., 2015), which report that parents are often not represented because the uncontrollability of the emergency somehow limits their function of protection for children. We may speculate that with the advent of the pandemic children may have perceived the uncertainty and the non-controllability of the situation even for the adults, and therefore they chose to represent the house that appeared to be the only secure place that could guarantee a certain degree of protection, stability and safety, also considering the age range of our participants. Future research in this direction is however auspicated to confirm our proposed explanation. For what concern the significative differences in comparing the three moments, as expected, the theme of the house, place of confinement, was largely illustrated in the “During” drawings that also include more details of the house compared to the “Before” and “Future” ones, reflecting the extremely high relevance of this context in the life of children during the pandemic due to social restrictions. It may be worth noting that adding details to the representation of the house is usually considered an index of care and therefore of a positive emotional investment in what is represented (Quaglia and Saglione, 1990). A significant difference in comparing the three moments, is also the major representation of friends in “Future” drawings, in line with our expected results and findings from other contemporary studies (Idoiaga Mondragon et al., 2021; Pascal and Bertram, 2021): this result may indicate a desire and a hope to return to spend time with friends, thus showing a certain trust in the future which is an important resource.

As far as concerns our third area of investigation, some relevant results emerge from the integration of quantitative and qualitative measures. The children that have more difficulties in taking into account the mental states of the others, as reported by parents, include their parents in “Before” drawings. This difficulty may cause them not to perceive the concern and the uncertainty of their caregiver, that therefore do not lose, in children’s perception, their function of protection and shelter. As already said before, in emergencies children often do not include their parents in drawings: in the face of events such as natural disasters or wars, parents are unable to maintain in the eyes of their children the safety function that they can usually perform, because they cannot control these events and also are tested by them (Giordano, 2012). Thus, children that are able in considering others’ mental states could perceive the worries, the uncertainty and probably also the difficulties of their parents in dealing with the COVID-19 pandemic, thus weakening to their eyes the function of protection and refuge of parents. However, we are aware that more future research is needed to confirm this claim. Moreover, children that include friends in “Before” drawing have fewer difficulties in the emotional area, as revealed by the SDQ. Including friends in a drawing of a past event, in the impossibility of spending time with them, at the time the drawings were collected, could be an emotional content too difficult to control for children that have difficulties in their emotional regulation. Indeed, other studies (Idoiaga Mondragon et al., 2021) highlight that children were sad, angry, upset and felt lonely because of the lack of friends. An unexpected result was the significant association between the exclusion of relatives in “Before” drawings and a higher level of difficulty in relationships with peers reported by parents. It could be hypothesized an influence caused by the presence of siblings in the coding label “Relatives.” Given the closure circumstances from which families came in the months prior to data collection, it was siblings the peers with whom parents were able to see their children relate with. As seen for other contents there was a tendency to exclude from drawings the elements of difficulty and discomfort. This appears a tendency especially for memories and hopes, whereas in representing lockdown experience some children also took the liberty of reporting a few unpleasant items (sad expressions, negative natural elements). In addition, the scores on the emotional subscale of SDQ were lower in children that included a happy facial expression in their “During drawing.” In considering happy facial expression it is important to keep in mind that there is a “mood bias” (Buckalew and Bell, 1985; Jolley et al., 2004), so that children, in general, tend to represent more happy expressions than sad ones.

Despite the interesting results reported so far, we are aware that this work is not without some limits. One of these is the reduced sample size, due to the difficulties in recruiting participants in such stressful period, and also the “privileged” population involved, which forces us to remain very cautious in generalizing the representations and contents that emerged. Another possible limitation is that we did not collect information about the level of familiarity, enjoyment and habit with the practice of drawing by the children involved. It would be desirable in subsequent studies to collect these data in order to insert considerations regarding the drawing of a potentially traumatic situation into the larger and more complete framework of how the child ordinarily knows, appreciates and uses the communicative tool of drawing. In addition, the results of this study may have been influenced by the particularity of the setting of administration. The fact that parents were asked to propose the activity to children was unavoidable, because of the restrictions imposed by the pandemic that prevented the researcher to collect data directly. Of course, this would have allowed us to gain more control over the possible variables in the setting, to collect more information and with a higher level of details that would have then allowed more specific reflection. However, involving parents actively in collecting children’s drawings and comments may have given children the opportunity to share emotions with their parents, and to build new meanings together with their reference figures, thus allowing a shared reflection of what they lived during the lockdown and of their hopes and desires for the future (Petrocchi et al., 2020; Prime et al., 2020; Bianco et al., 2021; Masten, 2021). Moreover, this work offered an occasion to discuss also the children’s representations of their parents that are generally absent in drawings.

As previously mentioned, using a qualitative research method requires a lot of caution in the interpretation and generalization of the collected data. Even if we consider the expressive indicators taken from the previous studies (Buckalew and Bell, 1985; Jolley et al., 2004; Picard et al., 2007; Brechet and Jolley, 2014; Misalidi and Bonoti, 2014; Rose and Jolley, 2020), they could be influenced by the age of the child or by the typical tendency to include more elements of positive connotation. Therefore, while maintaining the central focus on the perspective of children, it was also very relevant for us to collect, quantitatively, some information from parents. Also, the tasks proposed to parents could have had a first direct relapse in turning their attention and arouse in them a reflection on the behavior and experiences of their children. At the methodological level these additional data have made it possible to insert the graphic expression of children within a wider framework. Indeed, according to Brannen (2005), the combination of quantitative and qualitative research methods may result in confirmation, elaboration, complementarity, or contradiction, but in all cases, the combination leads to an integration and a more complete overview of the selected phenomena. Furthermore, the integration of qualitative and quantitative methods to collect, respectively, children’s and adults’ points of view could represent a precious source of information with the aim to highlight resources and needs of families, in prevision of potential interventions that could increase family wellbeing and coping skills. From this perspective, it could also be relevant to add information about parents’ experiences in order to relate these data, to what is communicated through the drawing by their children. A previous study showed that the level of distress of mothers may influence the attribution of negative emotions to children and their behaviors (Petrocchi et al., 2020). Therefore, it would be interesting observing and comparing the contents and emotions emerging from children’s drawings and, on the other hand, from mothers’ or parents’ representations. This addition would not be intended to reduce the centrality of the point of view of the child, but rather to maintain a vision as much as possible systemic and careful not to isolate the subject from its context, because children and parents are systems in continuous interaction with other external systems (Pianta, 2001).

## Conclusion

This study has provided an exploratory look at the experiences of children during the pandemic, thus allowing to have as much as possible authentic knowledge of the representations and the meanings aroused in children by the emergency linked to the pandemic.

The expression through the drawing and particularly the structure of the “Test de trois dessins: avant, pendant et avenir” (Crocq et al., 2005) could be an effective means for enabling children to express and share their experience of an event with exceptional nature, such as the pandemic. This also permitted us to explore the views of children which cannot be ignored given the challenge to their development that the pandemic has brought with it (Haleemunnissa et al., 2021). What seems to emerge is that the children of our sample have generally had sufficient resources to limit the potential negative impact on their development caused by the COVID-19 emergency. This does not mean that children have not perceived the concerns, changes and limitations that the pandemic has brought with it, for instance, the lacking of friendship relationships. The employment of a qualitative method like drawings allows understanding the point of view of the subject in all its complexity, specificity and richness. Indeed, with this type of instrument children are free to express their representation without the fear of being judged or evaluated. For this purpose, the choice, although forced, to delegate the administration to parents could have favored a more spontaneous and natural expression of their emotions, feelings and experiences, thus enriching the relationship between the child and the caregiver in such a complex period like the pandemic.

## Data availability statement

The raw data supporting the conclusions of this article will be made available by the authors, without undue reservation.

## Ethics statement

The studies involving human participants were reviewed and approved by the Ethical Committee for Research of the University of Bergamo. Written informed consent to participate in this study was provided by the participants’ or their legal guardian/next of kin.

## Author contributions

FB, GG, AM, DM, and IC conceptualized the study and supervised it. FB and AC were responsible for the data collection. GG and AC coded drawings. AC and DM analyzed the data. AC, FB, and IC wrote the draft manuscript. GG, AM, and DM provided feedback on the final manuscript. All authors read and approved the final version of the manuscript.

## Footnotes

1 During the first phase of COVID-19 health emergency in Italy “Everything will be ok” was the slogan that was reported with a drawing of a rainbow as a message of hope on billboards and banners hanging on windows and balconies.

2 This paper uses data based on the administration of only 23 questions. This is not permitted under the SDQ terms of use, which require users to administer authorized versions of the SDQ in full and without any modification (see Copyright Notice on https://www.sdqinfo.org/). The authors regret this error and commit to abide fully by the SDQ terms of use in future collection of SDQ data.
