# Corrigendum: Children's representations of the COVID-19 lockdown and pandemic through drawings

**DOI:** 10.3389/fpsyg.2024.1439212

**Published:** 2024-06-21

**Authors:** Alessia Cornaggia, Federica Bianco, Gabriella Gilli, Antonella Marchetti, Davide Massaro, Ilaria Castelli

**Affiliations:** ^1^Department of Psychology, Catholic University of Sacred Heart, Milan, Italy; ^2^Department of Human and Social Sciences, University of Bergamo, Bergamo, Italy

**Keywords:** children drawings, child development, COVID-19, emotions, relationship, mindreading abilities

In the published article, there was an error. In the study we used only 23 questions of the SDQ questionnaire due to the emergency period (i.e., 2 items were not applicable in a lockdown situation as we explain in the manuscript), but we have been contacted by the authors of the instrument, as our choice was not permitted within the Copyright Notice of their instrument. As required by the authors of SDQ, it is necessary to insert a note.

A correction has been made to *Materials and methods, Strength and difficulties questionnaire, Paragraph 1*. This sentence previously stated.

“In this work two items of the Peer Problems Scale were removed (“Picked on or bullied by other children” and “Generally liked by other children”), because in the specific period of the lockdown these aspects were not evaluable.”

The corrected sentence appears below.

“In this work two items of the Peer Problems Scale were removed (“Picked on or bullied by other children” and “Generally liked by other children”), because in the specific period of the lockdown these aspects were not evaluable.^2^

The following text has been added as Footnote 2.

^“2^This paper uses data based on the administration of only 23 questions. This is not permitted under the SDQ terms of use, which require users to administer authorized versions of the SDQ in full and without any modification (see Copyright Notice on https://www.sdqinfo.org/). The authors regret this error and commit to abide fully by the SDQ terms of use in future collection of SDQ data.”

The correspondence details of the original article have also been updated. The original email address provided has been replaced with the following: cornaggia13@gmail.com.

The authors apologize for this error and state that this does not change the scientific conclusions of the article in any way. The original article has been updated.

